# Personalized management of acenocoumarol dosage using eGFR: Analysis of INR data in a cohort of 204 patients 

**DOI:** 10.5414/CP204882

**Published:** 2025-10-09

**Authors:** Dzhem Farandzha, Vasil Velchev, Dobri Hazarbasanov

**Affiliations:** 1 Department of Cardiology, University Hospital St. Anna, Sofia, and; 2 Department of Cardiology, Heart and Brain Center of Clinical Excellence, Burgas, Bulgaria

**Keywords:** acenocoumarol dose, estimated glomerular filtration rate, CKD-EPI

## Abstract

Background: Acenocoumarol is a widely used vitamin K antagonist, particularly in European countries. While age and body metrics are known to influence dose requirements, the role of renal function in guiding acenocoumarol dosage remains underexplored. Aims: The primary aim of this study was to investigate the relationship between renal function and the weekly dose of acenocoumarol required to maintain therapeutic international normalized ratio (INR) levels. Materials and methods: We analyzed 425 INR measurements from 204 adult patients receiving acenocoumarol, stratified by target INR ranges of 2.00 – 3.00 and 2.50 – 3.50. Renal function was assessed using the body surface area (BSA)-adjusted chronic kidney disease-epidemiology (CKD-EPI) 2021 formula. Weekly acenocoumarol doses were evaluated in relation to age, sex, estimated glomerular filtration rate (eGFR), and body size. Results: Patients with lower eGFR and older age required significantly lower weekly doses of acenocoumarol. In the INR 2.00 – 3.00 group, males required higher doses than females, correlating with both greater body size and higher eGFR. However, in the INR 2.50 – 3.50 group, males and females received the same median dose despite differing body metrics, mirroring their similar renal function. A positive correlation was found between BSA-adjusted eGFR and weekly dose, particularly when eGFR exceeded 90 mL/min (Spearman r = 0.48, p = 0.0001). Conclusion: Renal function, as measured by BSA-adjusted eGFR, is a critical determinant of acenocoumarol dose requirements. These findings support the inclusion of renal function in future acenocoumarol dose calculators and emphasize the importance of individualized dosing strategies.

**What is known about this subject**

- It is known that acenocoumarol is widely used in some European countries as the only available vitamin K antagonist for oral anticoagulation [1, 2].
- It has been shown that age, body size, and genetics influence dose requirements of vitamin K antagonists [3, 4].
- It is a fact that renal function affects the pharmacokinetics of various medications, but data for acenocoumarol are limited [4, 5].

**What this study adds**

- We showed for the first time that BSA-adjusted renal function, as estimated by the CKD-EPI 2021 formula, significantly correlates with acenocoumarol dose in patients achieving therapeutic INR.
- We extended knowledge on real-world acenocoumarol dose requirements by identifying renal function as a more predictive variable than sex or body weight in specific patient subgroups.
- The clinical impact of our work is that dose individualization tools for acenocoumarol could be improved by incorporating eGFR alongside traditional variables.

## Introduction 

Acenocoumarol, a vitamin K antagonist (VKA) belonging to the coumarin class, is classically utilized for thromboembolic prevention and treatment. Unlike its counterpart, warfarin, acenocoumarol exhibits a much shorter half-life of 8 – 11 hours and is primarily metabolized by the liver, with subsequent renal elimination of its metabolites [5, 6]. Given the critical role of renal function in the elimination process, this study aims to investigate the potential impact of creatinine clearance on the dosage regimen of acenocoumarol, a factor that may be more significant than currently appreciated in clinical practice. 

## Material and methods 

This prospective observational study includes 204 individual patients on acenocoumarol therapy with a total of 425 laboratory measurements of international normalized ratio (INR), a universally recognized measure used to express the outcomes of the prothrombin time (PT) test [7, 8]. Simultaneously with the INR measurements, blood samples were collected to determine serum creatinine levels to assess kidney function. Patient enrollment took place at University Hospital Lozenetz, Sofia, Bulgaria, from November 2022 to January 2024, with additional outpatient measurements included up to February 2025. Participants were adults aged 18 years or older who had provided written informed consent and had been on acenocoumarol treatment for at least one month prior to enrollment without any dose adjustments in the week preceding sampling. Demographic and clinical data collected included age, sex, weight, height, and medical indications for acenocoumarol therapy, with patients classified into age groups as follows: 25 – 35, 36 – 45, 46 – 55, 56 – 65, 66 – 75, 76 – 85, and 86 years and above. The clinical indications for acenocoumarol use encompassed conditions such as venous thromboembolism (VTE), atrial fibrillation (AF), including AF associated with moderate to severe mitral stenosis of rheumatic origin, left ventricular thrombus (LVT), bioprosthetic heart valves (BHV), and thrombophilia among others with a target INR of 2.00 to 3.00. Patients with mechanical heart valves (MHV) were considered to have a therapeutic INR range of 2.50 to 3.50. The estimated glomerular filtration rate (eGFR) was calculated using the CKD-EPI formula for both indexed and non-indexed values from the National Kidney Foundation’s website [9, 10]. Statistical analysis was executed using GraphPad Prism 10, where descriptive statistics, group comparisons using Kruskal-Wallis tests, and correlations using Spearman correlation coefficients were evaluated since the data were not normally distributed [11]. 

## Results 

The cohort comprised 204 patients, with a distribution of 72 female and 132 male participants. The patient data included 425 laboratory measurements of INR. Out of the total INR samples, 264 were aimed to fall within the therapeutic range of 2.00 to 3.00. However, only half of these (132 samples) actually resided within this desired range, representing a 50% success rate in achieving the target INR for indications necessitating this level of anticoagulation. For the subgroup with MHV, where the target INR range was set higher at 2.50 to 3.50, 161 samples were analyzed, with only 53 (33%) meeting the target range. 

**Figure 1.**
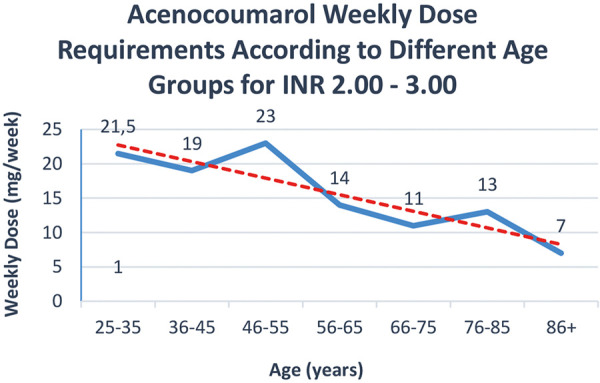
Weekly acenocoumarol dose requirements to reach therapeutic levels of INR within the range of 2.00 – 3.00. The dashed red tendency line clearly shows a decline in dose requirements with advancing age.

**Figure 2.**
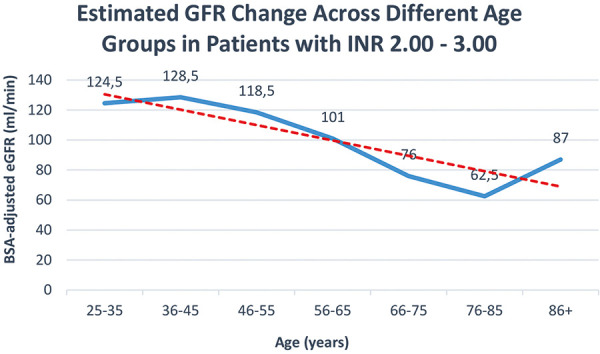
There is a decline in estimated glomerular filtration rate (eGFR) with advancing age in patients within the therapeutic INR range of 2.00 – 3.00.

Regarding sex differences in dosage among those requiring an INR between 2.00 to 3.00, the median weekly dose to reach therapeutic INR levels for females was 10 mg, notably lower compared to 16 mg for males. There were also physical disparities observed between the sexes; males exhibited a higher median body weight and greater stature compared to their female counterparts (89 vs. 66 kg and 175 vs. 160 cm, respectively). Despite these physical differences, the eGFR, calculated using the CKD-EPI 2021 formula, did not show a significant difference between males and females (84 vs. 83 mL/min/1.73m^2^, respectively). However, when eGFR was adjusted for body surface area, a notable divergence appeared, with males displaying significantly higher eGFR values (94 mL/min) compared to females (70 mL/min). The weekly acenocoumarol dose necessary to achieve therapeutic INR levels within the range of 2.00 – 3.00 exhibited a notable decline with increasing age (Figure 1). A similar tendency was observed with the BSA-adjusted eGFR (Figure 2). 

**Figure 3.**
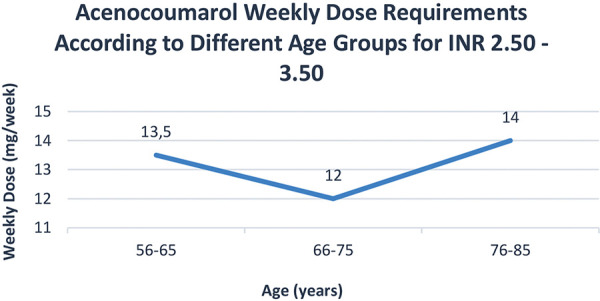
Acenocoumarol weekly dose requirements in patients with therapeutic levels of INR within the range of 2.50 – 3.50.

**Figure 4.**
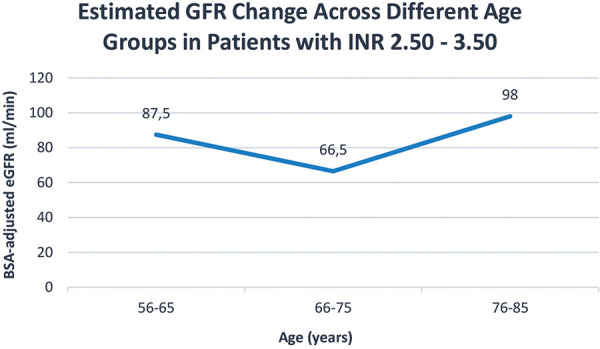
Estimated glomerular filtration rate (eGFR) in patients with therapeutic levels of INR within the range of 2.50 – 3.50. The line resembles the graph for the acenocoumarol weekly dose requirements above.

For the subgroup requiring a higher INR range of 2.50 to 3.50, out of 161 samples, only 53 (33%) met the target range. Interestingly, both sexes required the same median weekly dose of 12 mg to achieve this target INR, despite significant differences in weight (80 kg for males vs. 67 kg for females) and height (168 cm for males vs. 158 cm for females). The median creatinine levels were 102 mcmol/L for males and 77.5 mcmol/L for females, which although significantly higher in males, did not translate into a difference in renal function when assessed by the BSA-adjusted eGFR (75 mL/min for males and 74 mL/min for females), according to the CKD-EPI 2021 formula. The weekly dose requirements remained relatively consistent across age groups from 56 to 85 years, closely mirroring the trends in eGFR (Figure 3, Figure 4). 

**Figure 5.**
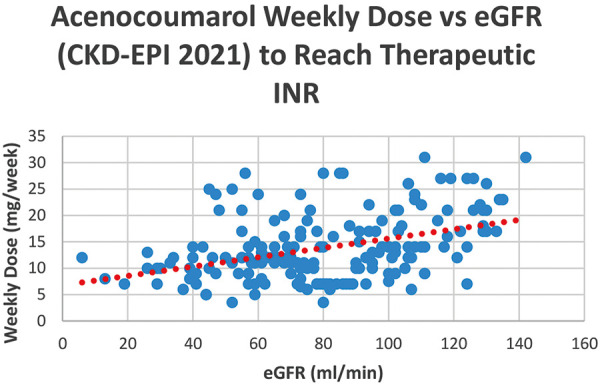
Scatter plot showing the distribution of weekly acenocoumarol dose and estimated glomerular filtration rate (eGFR) levels according to the BSA-adjusted CKD-EPI 2021 formula in all patients with therapeutic INR levels. The red dashed tendency line marks the positive relationship between eGFR and weekly acenocoumarol dose.

A statistically significant correlation was observed between eGFR values, as estimated by the BSA-adjusted CKD-EPI formula, and the weekly acenocoumarol dose with a Spearman r of 0.40 (CI, 0.27 to 0.52) and a two-tailed p-value of < 0.0001 (Figure 5). This correlation was even more pronounced when eGFR values exceeded 90 mL/min, showing a Spearman r of 0.48 (CI, 0.25 to 0.67) with a two-tailed p-value of 0.0001 for both INR target groups (2.00 – 3.00 and 2.50 – 3.50). 

**Figure 6.**
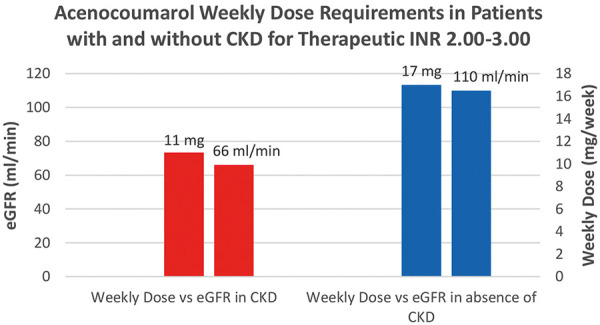
Column chart comparing weekly acenocoumarol dose requirements to reach therapeutic levels of INR within the range of 2.00 – 3.00 in patients with chronic kidney disease (CKD) (eGFR < 90 mL/min) and without CKD (estimated glomerular filtration rate (eGFR) ≥ 90 mL/min).

**Figure 7.**
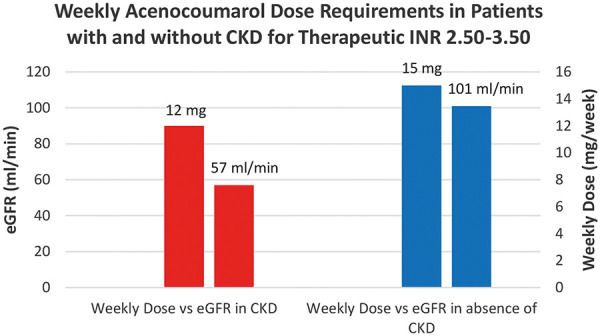
Column chart comparing weekly acenocoumarol dose requirements to reach therapeutic levels of INR within the range of 2.50 – 3.50 in patients with chronic kidney disease (CKD) (estimated glomerular filtration rate (eGFR) < 90 mL/min) and without CKD (estimated glomerular filtration rate (eGFR) ≥ 90 mL/min).

Consistent with initial expectations, the study found that patients with chronic kidney disease (CKD), characterized by an eGFR less than 90 mL/min, required lower doses of acenocoumarol to reach both INR ranges of 2.00 – 3.00 and 2.50 – 3.50 (Figures 6, Figure 7). There was no correlation between the weekly acenocoumarol dose and INR with Spearman rank correlation coefficient of 0,074 (95% CI, –0.02 to 0.17; p = 0.1266). 

## Discussion 

This study highlights significant findings on the role of renal function, body metrics, and age in determining the acenocoumarol dose necessary to achieve therapeutic INR levels. Our data suggest that while body weight and stature significantly influence dose requirements between sexes in the INR 2.00 – 3.00 group, renal function, as measured by BSA-adjusted eGFR, also plays a crucial role. Males required higher doses, which correlated not just with their greater body mass compared to females, but also with differences in renal function. 

Interestingly, in the INR 2.50 – 3.50 group, despite persistent weight and height differences between sexes, no significant differences in BSA-adjusted eGFR were observed, and both sexes required the same median weekly dose of acenocoumarol. This uniformity in dosage across sexes despite physical disparities underscores the predominant influence of renal function over body metrics in this therapeutic INR range. It aligns with our findings that suggest renal function adjustments are critical in acenocoumarol therapy, particularly for patients with varying degrees of renal health. 

The observed decrease in acenocoumarol dosage with advancing age across our study cohort supports the impact of diminishing renal function with age. Elderly patients typically exhibited lower eGFR values, necessitating reduced doses to avoid the risks of over-anticoagulation. This is consistent with previous studies that have identified age as a determinant factor in the pharmacokinetics and pharmacodynamics of anticoagulants [3, 12]. 

Our scatter plot analysis, which revealed a positive relationship between eGFR and weekly acenocoumarol dose across all patient groups achieving therapeutic INR levels, further solidifies the role of renal function in dosing decisions. This correlation was particularly pronounced at higher eGFR levels, highlighting the necessity to adjust doses based on precise renal function assessments. 

Acenocoumarol remains widely utilized in clinical practice across Europe and other regions, primarily due to lower costs compared to direct oral anticoagulants (DOACs) and familiarity within healthcare systems. Despite advantages offered by DOACs, such as fewer dietary interactions and reduced monitoring requirements, VKAs, including acenocoumarol, continue to be preferred in specific patient populations, notably those with mechanical heart valves, left ventricular assist devices (LVADs), antiphospholipid syndrome, patients with moderate-to-severe mitral stenosis and AF, significant renal impairment, or in resource-limited settings [13, 14, 15, 16, 17, 18]. Regular INR monitoring, necessary with acenocoumarol therapy, poses both logistical and patient compliance challenges, yet also offers opportunities for tailored dose adjustments. Patient preferences, availability of healthcare resources, and the clinical need for dose adjustments underscore the continued importance of research aiming to improve the safety and effectiveness of acenocoumarol therapy. 

## Conclusion 

This study provides compelling evidence that renal function, specifically BSA-adjusted renal function estimated by the CKD-EPI 2021 formula, significantly correlates with acenocoumarol dose requirements in patients achieving therapeutic INR levels, even when there are substantial differences in weight, height, and sex. Given that serious events related to anticoagulant therapy still occur, integrating renal function measurements into personalized dosing algorithms could enhance treatment safety, effectiveness, and overall patient outcomes. Future research should continue refining renal-function-based dosing strategies to address ongoing clinical challenges in anticoagulation management. 

## Acknowledgments 

The authors would like to thank all treating physicians for their role in data acquisition and providing informed consent to patients. 

## Authors’ contributions 

DF was involved in research conception, data acquisition, data analysis and manuscript preparation. VV contributed in data interpretation and revision of the article. DH was responsible for data analysis and critical revision of the article. 

## Funding 

The authors received no specific funding for this work. 

## Conflict of interest 

No conflict of interest.
